# Carboxyl-Functionalized, Europium Nanoparticle-Based Fluorescent Immunochromatographic Assay for Sensitive Detection of Citrinin in *Monascus* Fermented Food

**DOI:** 10.3390/toxins11100605

**Published:** 2019-10-17

**Authors:** Erjing Chen, Ying Xu, Biao Ma, Haifeng Cui, Chuanxin Sun, Mingzhou Zhang

**Affiliations:** 1 Zhejiang Provincial Key Laboratory of Biometrology and Inspection & Quarantine, China Jiliang University, Hangzhou 310018, China; s1709071002@cjlu.edu.cn (E.C.); s1809071028@cjlu.edu.cn (Y.X.); 16a0701109@cjlu.edu.cn (B.M.); hfcui@cjlu.edu.cn (H.C.); 2 Department of Plant Biology, Uppsala BioCenter, Linnean Centre for Plant Biology, Swedish University of Agricultural Science (SLU), P.O. Box 7080, SE-75007 Uppsala, Sweden

**Keywords:** Citrinin, Fluorescent immunochromatographic assay, Europium nanoparticles, *Monascus* fermented food

## Abstract

A fluorescent immunochromatographic test strip (FICTS) based on the use of europium nanoparticles (EuNPs) was developed and applied to detect citrinin (CIT) in *Monascus* fermented food. The sensitivity of the immunoassay to detect CIT was greatly improved by the use of a specific monoclonal antibody to attach EuNPs to form a probe. Under optimum conditions, the visual detection limit was 2.5 ng/mL, and the detection limit of the instrument was 0.05 ng/mL. According to the results, the IC_50_ was 0.4 ng/mL. Matrix interference from various *Monascus* fermented foods was investigated in food sample detection. The immunosensor also demonstrated high recoveries (86.8–113.0%) and low relative standard deviations (RSDs) (1.8–15.3%) when testing spiked *Monascus* fermented food. The detection results of this method showed a good correlation (*R^2^* > 0.98) with high-performance liquid chromatography (HPLC). The results showed that the FICTS method could be used as a rapid, sensitive method to detect CIT in *Monascus* fermented food.

## 1. Introduction

Citrinin (CIT), first isolated in 1931, is a hepato-nephrotoxic secondary metabolite synthesized from *Aspergillus* and *Penicillium* [1,2] and has been used as an experimental antibiotic in medical clinics [3]. However, research has shown that CIT commonly occurs in stored foods such as grains [4], fruits [5,6], and animal feeds [7,8], and CIT is also a secondary metabolite synthesized from *Monascus*. The production of commercially viable metabolites by *Monascus* species has been shown in many studies [9]. In China, the development of *Monascus* species has been widely used to include food colorants, cholesterol-lowering agents, and preservatives [10,11]. In Western countries, fermented products developed from *Monascus* are considered to be good health foods, which are loved and used to prevent cardiovascular disease. Many problems have gradually emerged with the widespread use of *Monascus* fermented food. The presence of CIT in fermentation products is a potential threat to public health [12]. Although it is evident that CIT exposure could exert toxic effects on the heart, liver, and kidneys, as well as the reproductive system, the genotoxic and mutagenic effects of CIT remain largely elusive [13]. According to the International Agency for Research on Cancer (IARC), there is insufficient scientific evidence to prove that CIT is carcinogenic. Accordingly, CIT is classified as a third type of carcinogen, and specific toxicity mechanisms of CIT have currently not been clearly reported [14]. This means that it is important to prevent and control CIT contaminants for safety reasons. Therefore, many countries have established statutory limits for CIT. The limit of citrinin in red fermented products in Japan is 200 μg/kg [15], and in the European Union it is 100 μg/kg. China has made a legal limit of 80 μg/kg for red yeast rice and its products [16].

In recent years, various chromatographic and mass spectrometric methods, such as liquid chromatography fluorescence (LC-FL) [17], high-performance liquid chromatography (HPLC) [18], ultra-performance liquid chromatography tandem mass spectrometry (UPLC-MS/MS) [19,20], ultra-performance liquid chromatography and fluorescence detection (UHPLC-FL) [21], gas chromatography, and selective ion monitoring (SIM) mass spectrometry (GC-MS) [22] have been well developed for detecting CIT content in *Monascus* fermented food. These protocols are exploited for accurate quantification and validation analyses because of their high accuracy and repeatability. However, they require complex operations, skilled personnel, sophisticated instruments, and time-consuming sample pretreatment; thus, they are unsuitable for rapid screening and on-site assays. Some microplate-based immunological methods have attracted amplified interest because of their high sample throughput, including enzyme-linked immunosorbent assay [23,24], antibody microarray immunoassay [25], and chemiluminescence immunoassay [26]. However, they cannot be used for field testing because they require multiple washing steps and long reaction times.

For decades, immunochromatographic test strip (ICTS) assays have been a popular screening tool for conducting on-site testing because of their sensitivity, rapidity, and ease of operation [27]. Colloidal gold is used as a metal nanoparticle-based probe that can be synthesized quickly and easily binds to antibodies [28]. Therefore, colloidal gold has been commonly used to detect mycotoxins such as ochratoxin A [29], fumonisin B1 [30], aflatoxin B1 [31], and zearalenene [32,33]. However, due to insufficient sensitivity, this label has not been applied to the detection of compounds at low concentrations. The advent of fluorescent signals has attracted widespread attention to enhance detection signals and increase the sensitivity of ICTS. Europium nanoparticles (EuNPs) offer a high sensitivity and long fluorescence lifetime. Simultaneously, they have no toxic effects on the sample. Therefore, EuNPs have been employed in food safety assays [34] and in medical diagnostics [35]. It has been reported that novel monoclonal antibodies against the avian influenza (AI) H7 subtype have been developed and applied to a EuNP-based rapid fluorescent immunochromatographic strip test to improve the sensitivity of the rapid diagnostic system. The sensitivity was improved 25-fold using europium, as confirmed by its comparison to colloidal gold-based rapid diagnostic kits [36]. Therefore, EuNPs used as labels could provide an excellent tool for improving the sensitivity of the ICTS assay.

In this paper, a competitive fluorescent immunochromatographic test strip (FICTS) assay was established for rapid quantitative determination of CIT at a high sensitivity. Moreover, the influence of various food matrix interference was also evaluated. The proposed method has the advantages of both the ICTS and EuNPs, enabling it to detect CIT in *Monascus* fermented food within 15 min. Experimental results demonstrated that the FICTS is capable of performing a rapid and sensitive quantitative analysis of CIT.

## 2. Results

### 2.1. Detection Principle

**Figure 1:**
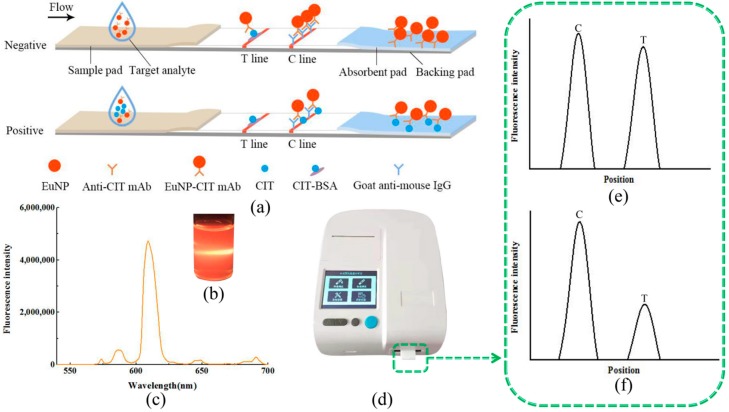
The detection principle of FICTS. (**a**) Schematic diagram of the FICST functioning: the sample is mixed with the anti-CIT mAb labeled with the EuNP and is applied to the strip. During their flow through the membrane, some EuNP-labeled mAbs bind to the CIT-BSA anchored at the Test line, while others reach the Control line, where are captured by a goat anti-mouse IgG. When CIT is present in the sample the binding of EuNP-labelled mAb at the Test line is inhibited. (**b**) Appearance of the strip under 365 nm irradiation. (**c**) Emission spectra of carboxylic acid functionalized EuNPs. (**d**) The FICTS reader (excitation/emission wavelengths of 365/610 nm). (**e**) Negative test result. (**f**) Positive test result.

The principle of the ICTS based on EuNPs for the detection of CIT is presented in Figure 1. First of all, the EuNP monoclonal antibody (mAb) probes were pre-mixed with sample solution containing CIT to form a uniform solution. Then, the sample solution was loaded onto the sample pad and diverted to the other end of the strip by capillary force. After reaction, the fluorescent signal intensities of strips were read and stored in the corresponding machine. There was competition between the homologous coating antigen and the target analyte for binding to the EuNP mAb probes. The EuNP mAb probes were able to combine with the target analytes specifically to form the EuNP mAb analyte complex, so the amount of EuNP mAb probes that could be captured by the test line was less than the original label amount, indicating a positive result (+) (Figure 1e). If the target analytes were absent in the sample solution, or lower than the detectable limit of the assay, all EuNP mAb probes would be captured by the homologous coating antigen on the test line. As the sample solution continued to migrate, the excess particles were captured by the secondary antibody on the control line. This phenomenon resulted in a negative result, as shown in Figure 1f. 

### 2.2. Identification of CIT Artificial Antigen and Monoclonal Antibody

The prepared citrinin–bovine serum albumin (CIT–BSA) and citrinin–human serum albumin (CIT–HSA) were identified by UV–vis spectra, respectively (Figure S1). The characteristic peaks of CIT were found at 250 and 310 nm. A characteristic peak of BSA was found at 275 nm. However, the characteristic peaks of the CIT–BSA were found at 278 and 320 nm. This above property may indicate that CIT was successfully coupled to the BSA carrier protein. In the same way, CIT–HSA was also successfully synthesized. 

The prepared CIT–BSA and CIT–HSA were identified by SDS-PAGE electrophoresis, respectively. The test results showed that the CIT–BSA band was found after the appearance of the BSA band, reflecting a hysteresis. It showed that the CIT was successfully coupled to BSA. This identification result was consistent with the UV scan identification results. The CIT–HSA band also reflected this characteristic, indicating that CIT was successfully coupled to HSA. The results are shown in Figure S2.

CIT–HSA was used as an immunogen to immunize mice. The spleen of immunized mice was fused with SP2/0 for cell fusion, and stable clones were obtained by subcloning. Subcloning showed the four stable cell lines, which were produced for ascites and purified by an octanoic acid–sulfur ammonium method to obtain monoclonal antibodies. Using CIT–BSA as a coating antigen, antibody affinity was determined using an indirect competitive ELISA (Figure S3) with four specific monoclonal antibodies: 2C3, 4B9, 7A1, and 9F6. We selected the best binding monoclonal antibody (4B9) and calculated its affinity to 5.17 × 10^8^ L/mol. Moreover, the antibody was evaluated using six different mycotoxins to show good specificity by indirect competitive ELISA (Table S1).

### 2.3. Optimization of FICTS Test Strip Parameters

As a rapid and sensitive immunoassay, the performance of the EuNP-based ICTS would be affected by some parameters, such as the concentration of labeled antibody, the immunoreaction time, different NC membrane options, the number of antibodies sprayed, and the number of coated films.

**Figure 2:**
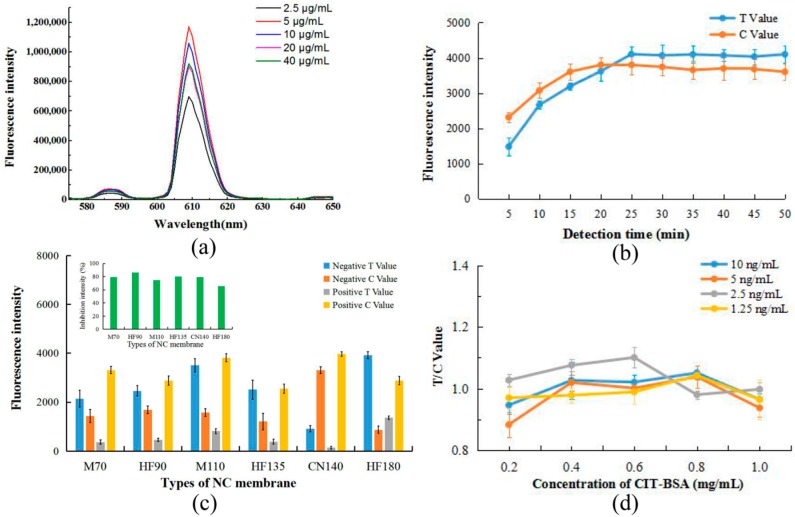
Optimization of FICTS assay. (**a**) The fluorescence intensity emitted by different antibody labeling amounts. (**b**) The fluorescence intensity analysis of different immunoreaction times. (**c**) The fluorescence intensity values and inhibition intensity were showed by using different nitrocellulose membranes. NC membrane types: M70 (MDI 70CNPH-N-SS40), HF90 (Millipore 90), M110 (MDI CNPF-SN12), HF135 (Millipore 135), CN140 (Sartorius CN140) and HF180 (Millipore 180). (**d**) Influence of different CIT–BSA concentrations and EuNP mAb probe concentrations on the fluorescence intensity.

As shown in Figure 2a, the increase in fluorescence intensity of the conjugation solution was not dependent on the increase in antibody concentration. When the amount of antibody labeled was 5 μg/mL, the fluorescence intensity was the highest. Therefore, the optimal concentration of anti-CIT mAb was 5 μg/mL.

Simultaneously, the effect of immunoreaction time on the fluorescence response of the biosensor was investigated. Figure 2b displays the fluorescent responses of the EuNP-based ICTS to different immunoreaction times, where the T value and the C value increased sharply with an increase in immunoreaction time within the first 15 min, and then it tended to gently increase until becoming stable at 25 min. Thus, 15 min was considered to be the optimal immunoreaction time, but 25 min could be considered the most complete immune response time.

Generally, the analytical performance of the FICTS was greatly affected by the nitrocellulose (NC) membrane. We tested NC membrane types HF135 (Millipore 135), M70 (MDI 70CNPH-N-SS40), HF90 (Millipore 90), M110 (MDI CNPF-SN12), CN140 (Sartorius CN140) and HF180 (Millipore 180). These NC membranes had no background fluorescence signal in UV visible light. The results are shown in Figure 2c. After comparisons with different NC membranes, we considered fluorescence intensity, inhibition intensity, and signal display effect. The NC membrane type Millipore 90 was proved to be the optimal choice for its low background noise, high fluorescence signal intensity, high sensitivity, and rapid testing.

To determine the best analytical performance of ICTS, the concentration of CIT–BSA coated on the NC membrane and the concentration of EuNP-labeled anti-CIT mAb sprayed on the glass fiber films were optimized. Cross-reaction experiments on the amount of antibody sprayed and the amount of coated on the film showed different results, as shown in Figure 2d. The amounts of coating antigen were 0.2, 0.4, 0.6, 0.8, and 1.0 mg/mL, respectively, and the amounts of antibody spray were 1.25, 2.5, 5, and 10 ng/mL, respectively. According to the results, considering the optimal display of the signal and the minimum amount of reagent, 0.6 mg/mL of CIT–BSA and 2.5 ng/mL of Eu-mAb were used as the optimum amounts in this study.

### 2.4. Comparison of FICTS and CG-ICTS

**Figure 3:**
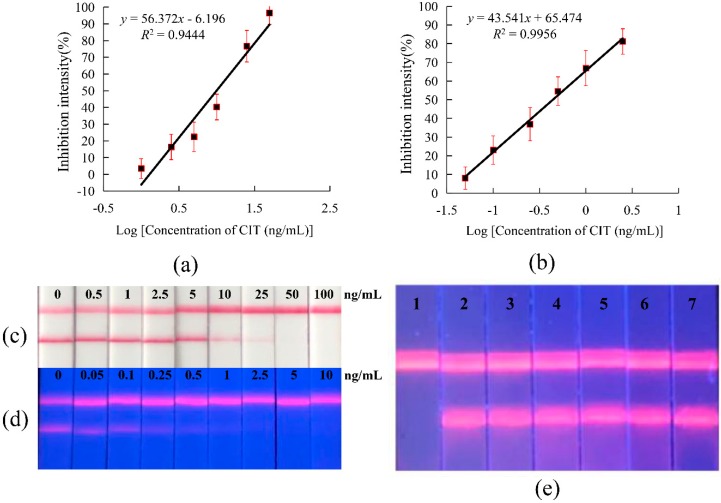
Detection in the FICTS assay and GC-LFIC assay. (**a**) The standard calibration curve for CIT by CG-ICTS. (**b**) The standard calibration curve for CIT by FICTS. (**c**) Sensitivity analysis of CIT by CG-ICTS. (**d**) Sensitivity analysis of CIT by FICTS. (**e**) Specific analysis of FICTS. 1: citrinin; 2–7: fumonisin B1, aflatoxins B1, vomitoxi, trichothecenes, zearalenone, and ochratoxin A.

CG-ICTS and FICTS assays were established based on the competitive form after system optimization. According to procedures reported in our laboratory, the CG-ICTS assay was established and optimized. The key optimization parameters were similar to FICTS. By adding a series of concentration standards, the sensitivity of strips was detected by the naked eye. The CG-ICTS and FICTS showed big differences. The color of the test line in the CG-ICTS measurement was eliminated at a standard concentration of 50 ng/mL, and the same line in the FICTS measurement almost disappeared at a standard concentration of 2.5 ng/mL. According to the values detected by the reader, the corresponding standard curves of CG-ICTS and FICTS are shown in Figure 3. The ratio of the T/C value of the 10% inhibition concentration to the T/C of the blank was defined as the limit of detection (LOD), and the ratio of the T/C of the 50% inhibition concentration to the T/C value of the blank was defined as IC_50_. Therefore, the CG-ICTS LOD was 1.9 ng/mL, and the IC_50_ was 9.9 ng/mL. The FICTS LOD was 0.05 ng/mL, and the IC_50_ was 0.4 ng/mL. The results indicated that the EuNPs were more suitable than traditional CG as a label in this study.

### 2.5. Specific Analysis of FICTS

To determine the specificity of the immunoassay, six mycotoxins, including fumonisin B1, aflatoxins B1, vomitoxi, trichothecenes, zearalenone, and ochratoxin A, were investigated. The six toxins were diluted to a standard solution at a final concentration of 500 ng/mL and were tested by the established FICTS assay. The results are shown in Figure 3e, indicating that the assay’s selectivity was consistent with the specificity of anti-CIT mAb, as measured by the ELISA technique (Table S1).

### 2.6. Matrix Interference in Real Samples

Matrix components, such as proteins and pigments, usually interfere with the FICTS assay. These components may cause a decrease in antibody affinity or non-specific absorption, which may affect the sensitivity of detection.

**Figure 4:**
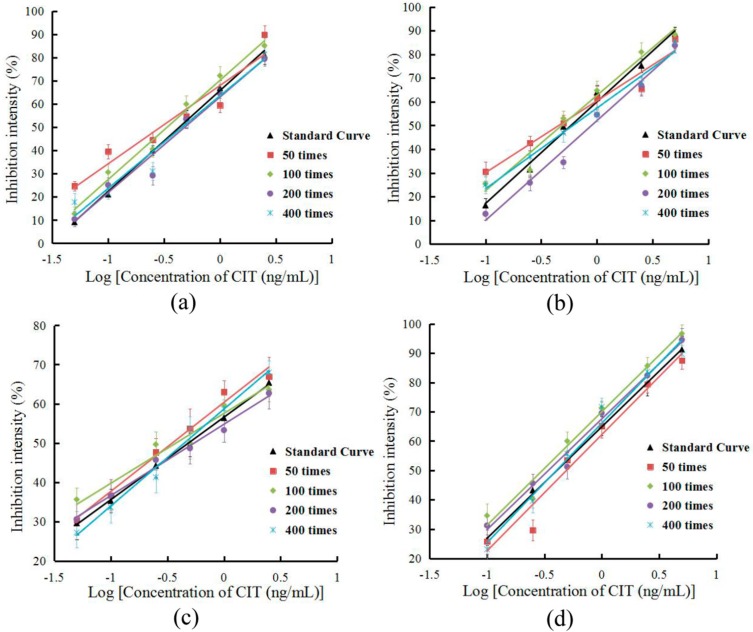
Fitted standard curve results of CIT at different dilution times in the real sample matrix. (**a**) Red yeast powder. (**b**) Red rice wine. (**c**) Red rice vinegar. (**d**) Rose fermented bean curd.

In this study, the effects of different *Monascus* fermented food substrates on the sensitivity of FICTS assays were evaluated. Dilution of the sample prior to detection eliminated matrix interference and ensured confidence in the method. The various sample extracts were diluted by 50, 100, 200, and 400 times with BBS buffer (0.05 M, pH 8.2) according to previous pretreatment methods. The standard solution was prepared from a series of sample buffers at different substrate concentrations. The results are shown in Table 1 and Figure 4. The matrix effect values of the four *Monascus* fermented food substrates were between 84.20% and 112.36% at 200-fold dilution. The results showed that matrix dilution could be ignored in the above dilution factor.

### 2.7. Detection of Spiked Samples by FICTS and HPLC

**Figure 5:**
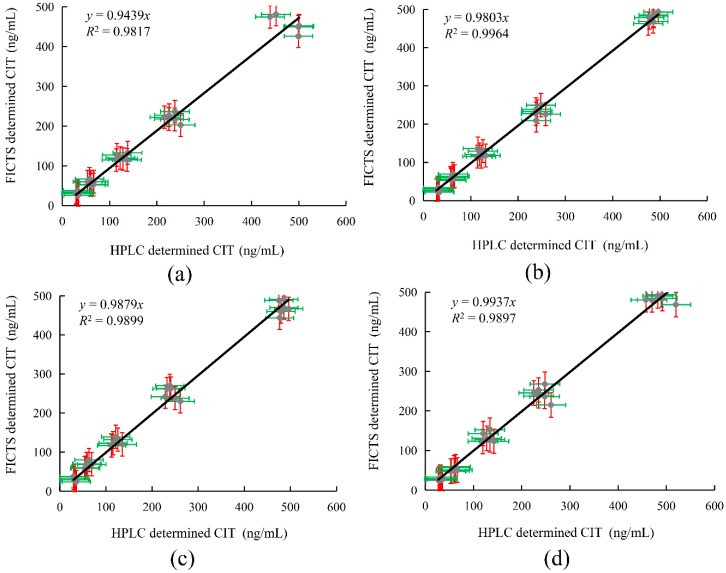
Correlation between FICTS and HPLC analyses of CIT in spiked *Monascus* fermented food. (**a**) Red yeast powder. (**b**) Red rice wine. (**c**) Red rice vinegar. (**d**) Rose fermented bean curd.

The accuracy of the FICTS assay was examined by detecting *Monascus* fermented food with different concentrations of CIT standard, and 6 replicates of each concentration were tested. The spiked samples were subjected to HPLC using the same criteria to determine the reliability of the FICTS analysis. According to the standard curves, the test values and average recovery rate were calculated. Furthermore, the precision and repeatability between batches could be reflected by the relative standard deviation (RSD). The results are shown in Table 2 and Figure 5. The recoveries of the spiked samples detected by FICTS were 86.8%~113.0%, and the relative standard deviations were less than 15.3%. The recoveries of spiked samples by HPLC were 96.8%~107.9%, and the relative standard deviations were less than 9.7%. Comparing the compatibility of the two methods, the correlation coefficient of the two methods was *R^2^* > 0.98, indicating that FICTS had a good correlation with HPLC and could be reliably applied to detect CIT in *Monascus* fermented food.

## 3. Discussion

Mycotoxins are natural toxins produced as secondary metabolites by fungi, especially *Aspergillus* and *Penicillium* species. They generally have carcinogenic characteristics and represent important contaminants in foods. CIT must be coupled to a protein carrier to exhibit this immunogenicity because it is a kind of small molecular weight mycotoxin, which is non-immunogenic. Therefore, we prepared artificial antigens to obtain specific monoclonal antibodies by linking CIT to BSA and HSA by the formaldehyde method [37]. The high sensitivity of the fluorescence immunoassay depends on the quality and classification of probes formed by monoclonal antibodies and fluorescent materials.

The development of fluorescence immunoassays has attracted wide attention. At present, more mature quantum dots have developed well in various detection fields. EuNPs have fewer reports. In this study, high-quality fluorescent probes were prepared from EuNPs and specific monoclonal antibodies. The addition of a fluorescent probe on the immunoassay strip can greatly improve the sensitivity in detecting CIT in *Monascus* fermented food.

Immunoassay strips have matured in the field of detection and have several advantages over other detection techniques, including short detection times, simple operation, and low cost. Operators without a technical background can use the immunoassay strip to obtain analytical results within ten minutes of the site. However, immunoassay strips have an insufficient sensitivity and cannot detect target objects at low concentrations. Fluorescence immunoassay test strips have the same basic advantages as immunoassay strips, and they only require a portable fluorescence detection machine to detect a lower concentration of CIT. The minimum detection limit for CIT in this method was 0.05 ng/mL, which is lower than the regulatory limit of China. Then, the comparisons of the analytical characteristics of this method and other methods for CIT are summarized in Table S2, which show that CIT was detected at a high sensitivity in this study. To reduce the impact of food matrices on the detection of fluorescent immunoassay strips, we analyzed the effects of food matrices. The results are shown in Table 1 and Figure 4. At a dilution factor of 200, strip detection was minimally affected by the influence of the food matrix. To confirm the validity and correctness of the analysis by using FICTS, we analyzed the recovery of CIT spiked into red yeast fermented food samples and compared it with HPLC. The results show that FICTS is a feasible method for detecting CIT in *Monascus* fermented food. In the future, the development and establishment of fluorescent immunoassay strips in various food testing fields will guarantee the safety of food. We will further study the method and improve it for real sample detection.

## 4. Conclusions

In this study, a FICTS immunoassay was successfully developed for the rapid detection of CIT, with good sensitivity, in *Monascus* fermented food samples. The assay could be completed in 15 min, and it was easy to operate. The FICTS immunoassay also showed a good linear fit (*R^2^* > 0.99) and high sensitivity for detecting citrinin more than CG-LCTS. The reliability of the immunoassay was verified by comparing the results of FICTS and HPLC. In summary, the described FICTS assay could be used to rapidly and accurately detect CIT in *Monascus* fermented food.

## 5. Materials and Methods

### 5.1. Reagents and Chemicals 

Fumonisin B1, aflatoxins B1, vomitoxi, trichothecenes, zearalenone, and ochratoxin A standards were purchased from the National Institute of Metrology, P. R. China (Beijing, China). Human Serum Albumin (HSA), bovine serum albumin (BSA), goat anti-mouse-HRP, Tween-20, dimethyl sulfoxide (DMSO), and Freund’s complete and incomplete adjuvants (cFA and iFA) were purchased from Sigma-Aldrich (St. Louis, MO, USA). 1-(3-Dimethylaminopropyl)-3-ethylcarbodiimide hydrochloride (EDC) was purchased from Aladdin Reagent Ltd. (Shanghai, China). 2-(N-morpholino) ethanesulfonic acid (MES) was supplied by Sangon Biotech Co., Ltd (Shanghai, China). Carboxylate-modified EuNPs with 200 nm diameter were purchased from Shanghai Uni Biotech Ltd (Shanghai, China). 3,3′,5,5′-Tetramethylbenzidine (TMB) was purchased from Dean Biological Co., Ltd. (Hangzhou, Zhejiang, China). RPMI 1640 cell culture medium, fetal bovine serum (FBS), HAT supplement, and HT supplement were purchased from Life Technologies Co. (Grand Island, NY, USA). The Sp2/0 myeloma cell line was obtained from the Chinese Academy of Sciences (Shanghai, China). Cell culture plates and 96-well microtiter plates were all purchased from Corning Inc. (Corning, NY, USA). Different types of NC membranes (Millipore 90, Millipore 135, Millipore 180, MDI 70CNPH-N-SS40, MDI CNPF-SN12, Sartorius CN140), glass fiber (used as sample and conjugate pads), and cotton pulp (used as an absorbent pad) were all obtained from Dean Biotechnology Co., Ltd. (Hangzhou, Zhejiang, China). Other conventional chemical reagents were purchased from Sinopharm Group Chemical Reagent Co., Ltd. (Shanghai, China).

The phosphate buffer solution (PBS, 0.01 M, pH 7.4) was obtained by mixing 0.19 g of KH_2_PO_4_, 0.19 g of KCl, 8.01 g of NaCl, and 2.30 g of Na_2_HPO_4_·12H_2_O in 1000 mL of ultrapure water with pH adjusted to 7.4. Borate buffer solution (BBS, 0.05 M, pH 8.2) was prepared by mixing 0.81 g of Boric acid and 0.67 g of NaB_4_O_7_·10H_2_O in ultrapure water in 1000 mL of ultrapure water with pH adjusted to 8.2. The preservation solution was prepared by adding 0.1% BSA (*w*/*v*) to BBS. MES buffer (0.05M, pH 6.5) was prepared by mixing 9.76 g of MES with 1000 mL of ultrapure water with pH adjusted to 6.5. All other reagents were of analytical grade and obtained from Sinopharm Chemical Corp. (Shanghai, China).

### 5.2. Apparatus

An XYZ3000 dispensing platform and CM2000 guillotine cutter (BioDot, Irvine, CA, USA) were used to prepare test strips. The Hitachi F-4500 fluorescence spectrometer system (Hitachi, Tokyo, Japan) was used to record the fluorescent spectrum. The FIC-S2011-B14 fluorescent strip reader (Suzhou Helmen Precise Instruments, Suzhou, Jiangsu, China) was used to scan strip results. The immunochromatographic strip results were compared with an Agilent 1100 high-performance liquid chromatography system (Agilent Tech, Santa Clara, CA, USA).

### 5.3. Preparation of CIT–BSA and CIT–HSA

The complete antigens were prepared according to Wu et al. [37], with some modifications using formaldehyde. CIT–HSA and CIT–BSA were used as the immunogen and coating antigen, respectively. Briefly, 5.0 mg CIT was dissolved in 0.5 mL methanol (100%), then formaldehyde (37%) was added. After 2 h reaction at 25 °C, 1.0 mg BSA was added. The mixture was incubated at 25 °C for 24 h. The final conjugates were dialyzed at 4 °C for 72 h against PBS buffer (0.01 M, pH 7.4). The collected samples were stored at −20 °C until use. CIT–HSA was synthesized by the same method.

### 5.4. Production of Monoclonal Antibody 

The “Administrative Rules for experimental animals in Zhejiang Province” (2009) were strictly complied with in this study to minimize animals’ suffering. Three Balb/c female mice were purchased from the laboratory animal center of Hangzhou Normal University (Hangzhou, China).

Referring to other previous studies, antibodies were produced in Balb/c mice [38]. Briefly, three Balb/c mice were immunized by subcutaneous injection of CIT–HSA and emulsified with an equal volume of Freund’s complete and incomplete adjuvant. After three subsequent injections, splenocytes of the immunized mice were taken out and fused with SP2/0 myeloma cells. The hybridomas secreting CIT monoclonal antibodies were screened using HAT and HT media. Then, the selected hybridomas were cloned and expanded into Balb/c mice to produce ascites containing CIT monoclonal antibody that could recognize CIT. Purification of ascites was done to obtain specific monoclonal antibodies (more details are available in the Supplementary Material).

### 5.5. Preparation of Colloidal Gold-mAb

According to the procedures used in our previous work [38], colloidal gold (CG) nanoparticles were prepared with an average diameter of 20 nm and coupled with the same specific mAb.

### 5.6. Preparation of EuNP mAb Probes

The conjugation process is related to that reported elsewhere [36]. One milligram of carboxylic EuNPs was dissolved in 400 μL MES (0.05M, pH 6.5) solution, then activated by adding 15 μL of EDC (10 mg/mL). After 30 min of incubation with slow shocking at room temperature (RT), the activated solution was centrifuged at 13,000 rpm for 30 min to separate EDC and dissolved in 500 μL of BBS (0.05 M, pH 8.2) by ultrasonic shock (100 W, 1 min). Afterward, 1 mL of anti-CIT mAb at concentrations of 2.5, 5, 10, 20, and 40 µg/mL was added and stirred gently at RT for 2 h. At the end of the reaction, 55 µL of 10% BSA (*w*/*v*) was added as a blocking buffer for 2 h. The EuNP mAb conjugates were centrifuged two times at 13,000 rpm to separate unreacted antibodies and BSA. Finally, the precipitates were resuspended in 500 μL of the preservation solution (0.05 M, pH 8.2) and stored at 4 °C until further use.

### 5.7. Preparation of the Test Strip

The ICTS assay consisted of five elements: sample pad, conjugate pad, NC membrane, absorbent pad, and backing card. Sample pads and conjugate pads made of glass fibers of the same specification were saturated with PBS buffer (0.01M, pH 7.4). The pads were placed in a desiccator at 37 °C for 2 h and stored in a sealed bag at RT before use. CIT–BSA and goat anti-mouse IgG (1.5 mg/mL) were applied to the NC membrane at a volume of 0.8 µL/cm and used as the test line and the control line, respectively. The test and control lines were spaced approximately 0.3 cm apart. The absorption pad was stored at RT without any treatment. All of the above components were assembled on a backing card, and each component overlapped 2 mm to ensure the solution migrated through the strip during the assay. Then, the entire assembled panel was cut into 2.5 mm strips and stored at RT.

### 5.8. FICTS Detection Procedure

For the CG-ICTS, 50 μL standard or sample solution was dropped on the sample pad, and the result was directly observed within the appropriate immunoreaction time. Simultaneously, the result was obtained by reading the optical response with the strip reader. For the FICTS, 50 μL standard or sample solution, EuNP mAb probes at a specific amount, and BBS buffer were mixed together and dropped onto the sample pad. Then, the results were observed within the appropriate immunoreaction time at 365 nm under a portable UV lamp. Simultaneously, the test strips were placed into the test strip reader, followed by recording of the fluorescence intensity of the test line and control line.

### 5.9. Standard Curves and Specificity

Under optimized conditions, the CIT standard solution was diluted in BBS buffer (0.05M, pH 8.2) at 0.05, 0.1, 0.25, 0.5, 1, 2.5, 5, and 10 ng/mL. A standard CIT curve was established by using a fluorescence reader. The IC_50_ and detection limit served as quantitative criteria to evaluate the performance of the FICTS assay.

To evaluate the specificity of the ICTS test, we tested several different compounds such as fumonisin B1, aflatoxin B1, vomitoxin, trichothecenes, zearalenone, and ochratoxin A. The mycotoxin standard solutions were diluted to a final concentration of 500 ng/mL, as described above, and tested individually.

### 5.10. Sample Analysis

To verify the applicability of the FICTS assay, spiked *Monascus* fermented food samples were analyzed by HPLC and FICTS, respectively.

First, the HPLC assay was performed according to national standards to identify the presence or absence of CIT residues in *Monascus* fermented food samples. The mobile phase consisted of acetonitrile (solvent A) and 0.1% phosphate (solvent B). The parameters were set as follows: flow rate 0.7 mL/min, C_18_ column (150 mm × 4.6 mm, 5 μm), temperature 30 °C, and injection volume 50 µL. The detection of CIT was performed with excitation/emission wavelengths of 350/500 nm, and the retention time was 9.1 min. The spiked samples were prepared by adding different concentrations of CIT (30, 60, 120, 240, and 480 ng/mL) to the negative samples.

The sample extraction method was similar to that of the above method [39]. Different concentrations of CIT standard were added to 1 g of the ground sample and then mixed with 20 mL of methanol:water (7:3, *v*/*v*) solution. The mixture was heated to 65 °C for 30 min and shaken for 30 s every 10 min. It was filtered through a glass fiber filter to clarify, and the filtrate was collected. One milliliter of the filtrate was taken and diluted with 39 mL of PBS solution (0.01M, pH 7.4) or more. The previous dilution was filtered through a glass fiber filter and collected in a clean container. All samples were analyzed by FICTS and confirmed by HPLC analysis.

## Figures and Tables

**Table 1:** Matrix effect of *Monascus* fermented food samples.

Sample	Dilution	Standard Curve	*R^2^*	IC_50_ (ng/mL)	Matrix Effect
Red yeast powder	50	*y* = 39.57*x* + 62.13	0.97	0.49	121.12%
	100	*y* = 38.63*x* + 69.93	0.98	0.31	74.70%
	200	*y* = 37.70*x* + 67.49	0.99	0.34	84.20%
	400	*y* = 40.68*x* + 66.03	0.98	0.40	98.95%
Red rice wine	50	*y* = 34.20*x* + 68.65	0.99	0.29	67.84%
	100	*y* = 42.77*x* + 70.21	0.99	0.35	77.90%
	200	*y* = 41.14*x* + 62.97	0.97	0.48	111.75%
	400	*y* = 39.89*x* + 63.52	0.96	0.46	105.81%
Red rice vinegar	50	*y* = 30.31*x* + 60.38	0.96	0.46	77.57%
	100	*y* = 40.23*x* + 62.69	0.98	0.48	82.54%
	200	*y* = 18.16*x* + 54.74	0.99	0.52	88.35%
	400	*y* = 34.05*x* + 57.32	0.99	0.61	103.96%
Rose fermented bean curd	50	*y* = 22.67*x* + 60.32	0.99	0.35	71.82%
	100	*y* = 17.81*x* + 57.51	0.95	0.38	77.57%
	200	*y* = 18.16*x* + 54.74	0.99	0.55	112.36%
	400	*y* = 24.68*x* + 58.61	0.99	0.45	91.81%

**Table 2:** Recovery rate determination of CIT in *Monascus* fermented food by FICTS with HPLC.

Sample	Spiked Level (ng/mL)	FICTS			HPLC		
		Detected Amount (ng/mL)	Recovery Rate	RSD (*n* = 6)	Detected Amount (ng/mL)	Recovery Rate	RSD (*n* = 6)
Red yeast powder	30	31.8	106.0%	12.7%	31.6	105.3%	7.5%
	60	58.9	98.2%	9.4%	60.7	101.1%	8.2%
	120	121.2	101.0%	6.1%	125.9	104.9%	8.2%
	240	220.6	91.9%	5.1%	232.2	96.8%	5.2%
	480	455.6	94.9%	4.3%	481.6	100.3%	5.9%
Red rice wine	30	27.5	91.8%	14.6%	30.8	102.5%	7.7%
	60	60.8	101.3%	9.9%	61.0	101.7%	5.5%
	120	122.4	102.0%	7.1%	121.1	100.9%	5.5%
	240	230.6	96.1%	5.9%	243.7	101.5%	3.4%
	480	478.3	99.7%	2.4%	484.4	100.9%	1.6%
Red rice vinegar	30	30.8	102.7%	15.3%	32.0	106.7%	7.0%
	60	67.8	113.0%	11.3%	59.9	99.7%	9.3%
	120	124.4	103.7%	7.4%	120.5	100.4%	7.6%
	240	250.6	104.4%	6.5%	241.9	100.8%	5.0%
	480	469.9	97.9%	3.8%	483.2	100.7%	1.6%
Rose fermented bean curd	30	28.9	96.4%	9.7%	31.3	104.3%	9.7%
	60	52.1	86.8%	9.2%	59.3	98.8%	9.0%
	120	133.0	110.8%	8.9%	129.4	107.9%	7.0%
	240	242.4	101.0%	7.3%	241.9	100.8%	5.2%
	480	482.7	100. 6%	1.8%	485.4	101.1%	4.4%

## Supplementary Materials

The following are available online at https://www.mdpi.com/2072-6651/11/10/605/s1, Figure S1: UV spectrum of the CIT artificial antigens. Figure S2: SDS-PAGE electrophoresis of CIT artificial antigens. Figure S3: Affinity identification of different CIT monoclonal antibodies. Table S1: Cross-reactivity of anti-CIT monoclonal antibody with CIT and other mycotoxins Table S2. Comparison of the proposed method for CIT detection with other methods.

## References

[1] Bennett J.W., Klich M. (2013). Mycotoxins. Clin. Microbiol. Rev..

[2] Chu F.S. (1991). Mycotoxins: Food contamination, mechanism, carcinogenic potential and preventive measures. Mutat. Res..

[3] Hetherington A.C., Raistrick H. (1931). Studies in the biochemistry of micro-organisms. XIV. On the production and chemical constitution of a new yellow colouring mater, citrinin, produced from glucose by Penicillium citrinum, Thom. Philos. Trans. R. Soc. Ser. B.

[4] Pleadin J., Zadravec M., Lešić T., Vahčić N., Frece J., Mitak M., Markov K. (2018). Co-occurrence of ochratoxin a and citrinin in unprocessed cereals established during a three-year investigation period. Food Addit. Contam. B..

[5] Cheng H.W., Yang Y., Chen Y.F., Chen X.Q., Cai Z.Z., Du A.F. (2018). Novel monoclonal antibody-based immunochromatographic strip for detecting citrinin in fruit from zhejiang province, china. PLoS ONE.

[6] Ostry V., Malir F., Cumova M., Kyrova V., Toman J., Grosse Y., Pospichalova M., Ruprich J. (2018). Investigation of patulin and citrinin in grape must and wine from grapes naturally contaminated by strains of, *Penicillium expansum*. Food Chem. Toxicol..

[7] Visconti A., Bottalico A. (1983). High levels of ochratoxins a and b in moldy bread responsible for mycotoxicosis in farm animals. J. Agric. Food Chem..

[8] Arroyo-Manzanares N., Rodríguez-Estévez V., Arenas-Fernández P., García-Campaña A.M., Gámiz-Gracia L. (2019). Occurrence of Mycotoxins in Swine Feeding from Spain. Toxins.

[9] Wang T.H., Lin T.F. (2007). Monascus rice products. Advances in Food and Nutrition Research.

[10] Li Y., Zhou Y.-C., Yang M.-H., Ou-Yang Z. (2012). Natural occurrence of citrinin in widely consumed traditional chinese food red yeast rice, medicinal plants and their related products. Food Chem..

[11] Li F., Xu G., Li Y., Chen Y., Ji R. (2005). Natural occurrence of citrinin in Monascus products. Wei Sheng Yan Jiu (J. Hyg. Res.).

[12] Liu B.H., Wu T.S., Su M.C., Chung C.P., Yu F.Y. (2005). Evaluation of citrinin occurrence and cytotoxicity in Monascus fermentation products. J. Agric. Food Chem..

[13] de Oliveira Filho J.W.G., Islam M.T., Ali E.S., Uddin S.J., Santos J.V.O., de Alencar M.V.O.B., Júnior A.L.G., Paz M.F.C.J., de Brito M.D.R.M., E Sousa J.M.C. (2017). A comprehensive review on biological properties of citrinin. Food Chem. Toxicol..

[14] IARC (1986). Some Naturally Occurring and Synthetic Food Components, Furocoumarins and Ultraviolet Radiation. Monographs on the Evaluation of the Carcinogenic Risk of Chemicals to Humans.

[15] Liao C.D., Chen Y.C., Lin H.Y., Chiueh L.C., Shih D.Y.C. (2014). Incidence of citrinin in red yeast rice and various commercial Monascus products in Taiwan from 2009 to 2012. Food Control..

[16] GB.5009.222-2016 (2017). National Food Safety Standards for Determination of Citrinin in Monascus Products.

[17] Marley E., Brown P., Leeman D., Donnelly C. (2016). Analysis of citrinin in cereals, red yeast rice dietary supplement, and animal feed by immunoaffinity column cleanup and lc with fluorescence detection. J. AOAC Int..

[18] Vrabcheva T., Usleber E., Dietrich R., Märtlbauer E. (2000). Co-occurrence of ochratoxin A and citrinin in cereals from Bulgarian villages with a history of Balkan endemic nephropathy. J. Agric. Food Chem..

[19] Liu Y.Q., Wang H.W., Yao S., Zhu P.J. (2018). Detection of trace amounts of citrinin in dried orange peel by using an optimized extraction method coupled with ultra-performance liquid chromatography-tandem mass spectrometry. Biomed. Chromatogr..

[20] Wei F., Liu X., Liao X., Shi L., Zhang S., Lu J., Zhou L., Kong W. (2019). Simultaneous determination of 19 mycotoxins in lotus seed using a multimycotoxin UFLC-MS/MS method. J. Pharm. Pharmacol..

[21] Huertas-Pérez J.F., Arroyo-Manzanares N., García-Campaña A.M., Gámiz-Gracia L. (2015). High-throughput determination of citrinin in rice by ultra-high-performance liquid chromatography and fluorescence detection (UHPLC-FL). Food Addit. Contam. Part A.

[22] Shu P.-Y., Lin C.-H. (2002). Simple and sensitive determination of citrinin in Monascus by GC-selected ion monitoring mass spectrometry. Anal. Sci..

[23] Yirga S.K., Ling S.M., Yang Y.L., Yuan J., Wang S.H. (2017). The preparation and identification of a monoclonal antibody against citrinin and the development of detection via indirect competitive elisa. Toxins.

[24] Zhang C.X., Zhang Q., Tang X.Q., Zhang W., Li P.W. (2019). Development of an anti-idiotypic VHH antibody and toxin-free enzyme immunoassay for ochratoxin A in cereals. Toxins.

[25] Zhang X., Wang Z.H., Fang Y., Sun R.J., Cao T., Paudyal N., Fang W.H., Song H.H. (2018). Antibody microarray immunoassay for simultaneous quantification of multiple mycotoxins in corn samples. Toxins.

[26] Kim S., Lim H.B. (2015). Chemiluminescence immunoassay using magnetic nanoparticles with targeted inhibition for the determination of ochratoxin A. Talanta.

[27] Vanrell L., Gonzalez-Techera A., Hammock B.D., Gonzalez-Sapienza G. (2013). Nanopeptamers for the development of small-analyte lateral flow tests with a positive readout. Anal. Chem..

[28] Wang Z., Zhi D., Zhao Y., Zhang H., Wang X., Ru Y., Li H. (2014). Lateral flow test strip based on colloidal selenium immunoassay for rapid detection of melamine in milk, milk powder, and animal feed. Int. J. Nanomed..

[29] Liu B.H., Tsao Z.J., Wang J.J., Yu F.Y. (2008). Development of a monoclonal antibody against ochratoxin A and its application in enzyme-linked immunosorbent assay and gold nanoparticle immunochromatographic strip. Anal. Chem..

[30] Wang Y.K., Shi Y.B., Zou Q., Sun J.H., Chen Z.F., Wang H.A., Li S.Q., Yan Y.X. (2013). Development of a rapid and simultaneous immunochromatographic assay for the determination of zearalenone and fumonisin B1 in corn, wheat and feedstuff samples. Food Control..

[31] Wang S., Quan Y., Lee N.A., Kennedy I.R. (2006). Rapid determination of fumonisin B 1 in food samples by enzyme-linked immunosorbent assay and colloidal gold immunoassay. J. Agric. Food Chem..

[32] Xu Y., Huang Z.B., He Q.H., Deng S.Z., Li L.S., Li Y.P. (2010). Development of an immunochromatographic strip test for the rapid detection of deoxynivalenol in wheat and maize. Food Chemistry..

[33] Huang Z.B., Xu Y., Li L.S., Li Y.P., Zhang H., He Q.H. (2012). Development of an immunochromatographic strip test for the rapid simultaneous detection of deoxynivalenol and zearalenone in wheat and maize. Food Control..

[34] Majdinasab M., Zareian M., Zhang Q., Li P. (2019). Development of a new format of competitive immunochromatographic assay using secondary antibody–EuNPsropium nanoparticle conjugates for ultrasensitive and quantitative determination of ochratoxin A. Food Chem..

[35] Nankoberanyi S., Mbogo G.W., LeClair N.P., Conrad M.D., Tumwebaze P., Tukwasibwe S., Kamya M.R., Tappero J., Nsobya S.L., Rosenthal P.J. (2014). Validation of the ligase detection reaction fluorescent microsphere assay for the detection of Plasmodium falciparum resistance mediating polymorphisms in Uganda. Malar. J..

[36] Yeo S.J., Bao D.T., Seo G.E., Bui C.T., Kim H., Anh N.T.V., Trinh T., Nguyen L., Sohn H.J., Sohn C.K. (2017). Improvement of a rapid diagnostic application of monoclonal antibodies against avian influenza h7 subtype virus using EuNPsropium nanoparticles. Sci. Rep..

[37] Wu S.W., Yu Y.A., Liu B.H., Yu F.Y. (2018). Development of a sensitive enzyme-linked immunosorbent assay and rapid gold nanoparticle immunochromatographic strip for detecting citrinin in monascus fermented food. Toxins.

[38] Gong Y.F., Zhang M.Z., Wang M.Z., Chen Z.L., Xi X. (2014). Development of immuno-based methods for detection of melamine. Arab. J. Sci. Eng..

[39] Kong D., Xie Z., Liu L., Song S., Kuang H. (2017). Development of ic-elisa and lateral-flow immunochromatographic assay strip for the detection of citrinin in cereals. Food Agric. Immunol..

