# Supplementary material for: Carboxyl-Functionalized, Europium Nanoparticle-Based Fluorescent Immunochromatographic Assay for Sensitive Detection of Citrinin in Monascus Fermented Food
Source: Toxins (Basel). 2019 Oct 17;11(10):605. doi: 10.3390/toxins11100605 (PMC6832703; doi:10.3390/toxins11100605)
Supplement: Supplementary file 1 [file toxins-11-00605-s001.pdf]

toxins

Supplementary Materials: Carboxyl-Functionalized Europium-Based Fluorescent Immunoassay for Sensitive Detection of Citrinin in Monascus Fermented Food

Erjing Chen, Ying Xu, Biao Ma, Haifeng Cui, Chuanxin Sun and Mingzhou Zhang

S1. Preparation and Identification of Citrinin Artificial Antigen

Citrinin (CIT) is a kind of small molecular weight mycotoxin which is non-immunogenic. Therefore, we prepared artificial antigens to obtain specific monoclonal antibodies by linking CIT to carrier protein [1]. To obtain evidence of successful conjugation, the UV–vis spectra and 12% SDS-PAGE were measured for BSA, HSA, CIT, CIT–BSA and CIT–HSA conjugate, as shown in Figure S1 and S2.

Figure S1a shows that the characteristic peak of CIT–BSA was apparently significantly different from the UV absorption characteristics of CIT and BSA. Specifically, the characteristic peaks of CIT were found at 250 nm and 310 nm, and the characteristic peak of BSA was found at 275 nm. However, the characteristic peaks of the CIT–BSA were found at 278 nm and 320 nm. This above property may indicate that CIT was successfully coupled to the BSA carrier protein. In the same scheme, different specific absorption peaks of CIT–HSA and CIT and BSA were successfully detected (Figure S1b). It was shown that CIT was successfully coupled to the HSA.

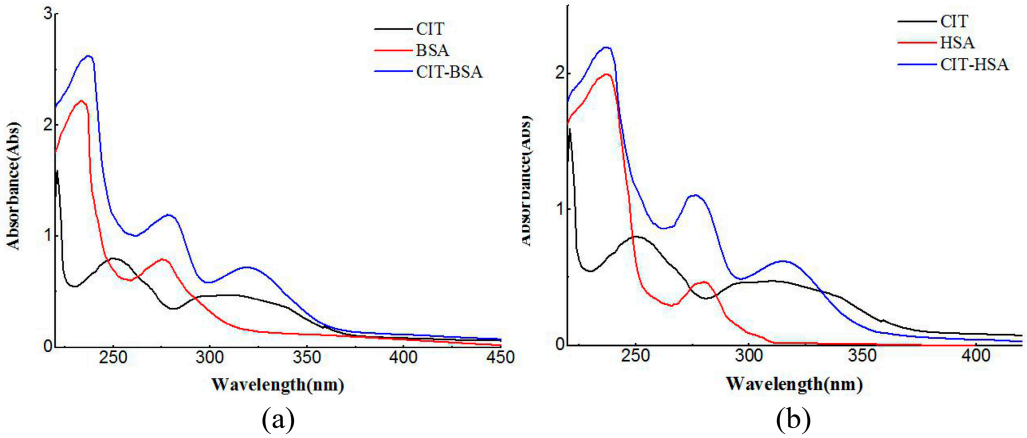

Figure S1. UV spectrum of the CIT artificial antigens. (**a**) UV spectrum scan of CIT–BSA. (**b**) UV spectrum scan of CIT-HSA.

Figure S2a indicates that the CIT–BSA band was found after the appearance of the BSA band, reflecting a significant hysteresis. Therefore, CIT successfully binds to the residues of BSA to form a conjugate. Significant hysteresis indicates that more CIT was attached to the BSA. This identification result was consistent with the UV–vis spectra identification results. In addition, the CIT–HSA strip also reflected this characteristic, indicating that CIT was successfully coupled to HSA (Figure S2b). However, the migration difference between CIT–HSA and HSA was much smaller than between BSA and CIT–BSA. This suggested that the CIT was less attached to the HSA than does CIT–BSA.

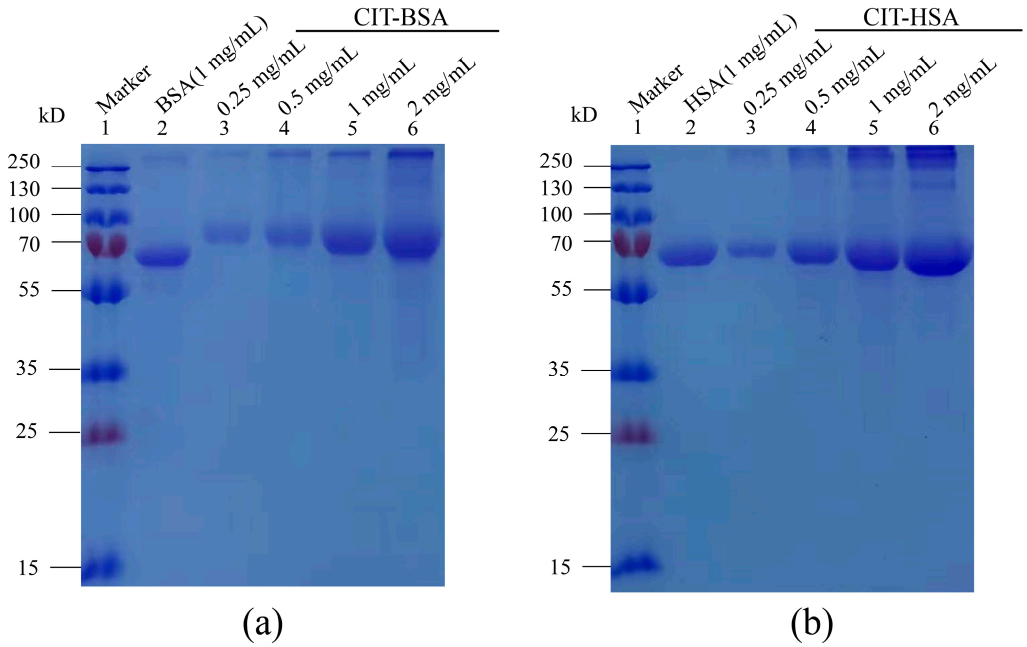

Figure S2. SDS-PAGE electrophoresis of CIT artificial antigens. (**a**) SDS-PAGE electropherogram of CIT–BSA. (**b**) SDS-PAGE electropherogram of CIT–HSA.

S2. Production of Monoclonal Antibody

The "Administrative Rules for experimental animals in Zhejiang Province" (2009) were strictly complied with in this study to minimize animals' suffering. Three Balb/c female mice were purchased from the laboratory animal center of Hangzhou Normal University (Hangzhou, China).

Referring to other previous studies, antibodies were produced in Balb / c mice [2]. Specifically, three Balb/c mice were immunized by subcutaneous injection of CIT-HSA (200 μg in 100 μL sterile PBS) and emulsified with an equal volume of Freund's complete adjuvant. For the subsequent immunizations (three times, at two-weekly intervals) 100 μg of CIT–HSA conjugate (in 100 μL sterile PBS) in the same volume Freunds incomplete adjuvant was used. Blood was obtained via retro-orbital venous plexus and stored at 4 °C overnight. Then, the segregated sera were examined for titer and inhibition ratio against free CIT by using indirect competition enzyme-linked immunosorbent assay, in which CIT–BSA was used as the coating antigen. When the antiserum titer no longer changed, the mice whose antiserum showed the strongest inhibition were further fused. Three days prior to spleen harvest, 100 μg of CIT–HSA was injected into the selected mice by spleen in 0.9% NaCl solution.

Murine myeloma cells Sp2/0 were maintained in an exponential growth stage in RPMI 1640 cell culture medium supplemented with 10% fetal bovine serum (FBS). The splenocytes from the immunized mice were fused with the myeloma cells. Hybridomas were selected in a HAT medium (DMEM medium containing 15% FBS). The cultures in 96-well plates were maintained in a 5% CO_2 incubator at 37 °C. The hybridoma that secreted antibodies specific to CIT were subcloned by the serial dilution method. Then the stable cells were expanded and cryopreserved in liquid nitrogen. The stable cells were produced for ascites and purified by octanoic acid-sulfur ammonium method to obtain monoclonal antibodies.

S3. Indirect Competitive ELISA

After checkerboard optimization, the indirect competitive ELISA was processed as the standard procedure for detection of antisera or identification of antibodies [3]. The detailed procedure was as follows. The 96-well plates coated with CIT–BSA were blocked at 4 °C for 24 h. After the reaction was completed, the 96-well plates were dried at 37 °C for 5 h and stored at 4 °C until use. PBS and diluted CIT standard were added to the closed 96-well plates at 50 μL/well. Then, anti-CIT monoclonal

antibody and goat anti-mouse-horseradish peroxidase were diluted and added to the closed 96-well plates at 50 μL/well. The reaction was carried out at 25 °C for 30 min. After the reaction was completed, the plates were washed with PBST for 3 times. Then, the TMB chromogenic reagent was added to the 96-well plates at 100 μL/well. The reaction was performed at 25 °C for 10 min. The stop solution was added to the plate at 50 μL/well, and the OD value was measured at 450 nm with a microplate reader.

S4. Identification of Anti-CIT Monoclonal Antibody

After the stable cells were produced for ascites and purified by octanoic acid-sulfur ammonium method to obtain monoclonal antibodies, the antibody affinities were determined using an indirect competitive ELISA (Figure S3). On account of the greater binding rates, the monoclonal antibody of clone 4B9 was selected for further use.

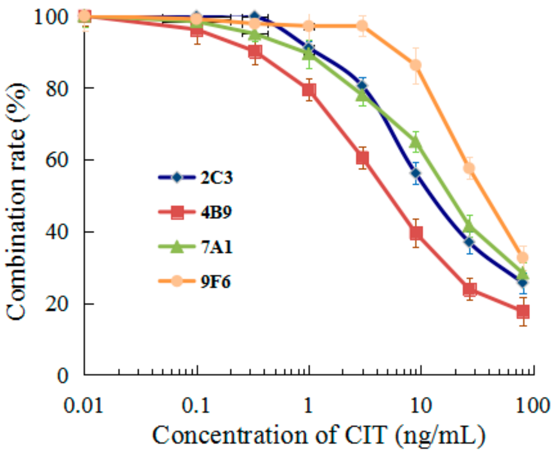

Figure S3. Affinity identification of different CIT monoclonal antibodies.

Cross-reactivity (CR) of anti-CIT monoclonal antibody with other mycotoxins would increase the interference of false positives. Thus, specificity of the antibody was evaluated using the six different mycotoxins by indirect competitive ELISA. The CR values were calculated as the following equation. CR (%) = [IC_{50} (CIT)/IC_{50} (compounds)] × 100%. The Table S1 was showed that the IC50 was 5.53 ± 0.76 ng/mL. According to calculation, the anti-CIT mAb had a CR of less than 0.1% for the other six different mycotoxins. The results showed that the anti-CIT mAb had almost no cross-reactivity to other six different mycotoxins. It could be seen that the anti-CIT mAb showed good specificity.

Table S1. Cross-reactivity of anti-CIT monoclonal antibody with CIT and other mycotoxins.

Mycotoxins	IC_{50} (ng/mL)	Cross-Reactivity (%)
Citrinin	5.53 ± 0.76	100
Fumonisin	>810	<0.1
Aflatoxins	>810	<0.1
Vomitoxi	>810	<0.1
Trichothecenes	>810	<0.1
Zearalenone	>810	<0.1
Ochratoxin A	>810	<0.1

S5. Supplementary Note to Table S2

For the detection of small molecular substances, the analysis characteristics of the whole method are reflected in the calibration cruves range, detection time and 50% inhibition (IC_{50}), limit of detection (LOD) and so on. Then the comparison of the analytical characteristics of this method and other

methods for CIT is summarized in Table S2, which showed that the high sensitivity of the present study detects CIT.

Table S2. Comparison of the proposed method for CIT detection with other methods**.**

Method	Calibration Cruves Range (μg/kg)	LOD (μg/kg)	IC_{50} (μg/kg)	Detection Time (min)	Reference
LC-FL	10–200	3.00	NR	25.00	[4]
GC-SIM	1–10	0.60	NR	10.89	[5]
LC-MS/MS	0.5–10	0.05	NR	NR	[6]
HPLC	0.1–1000	0.80	NR	NR	[7]
	1–100	NR	NR	6.00	[8]
	0.25–500	20.00	NR	10.40	[9]
UHPLC-FL	10–500	1.50	NR	5.00	[10]
ELISA	0–405	9.00	NR	NR	[11]
	2.5–10	5.00	NR	165.00	[12]
	0.01–5.96	0.01	0.28	315.00	[13]
	0.1–10	0.09	0.76	315.00	[14]
CG-ICTS	0–50	50.00	NR	5.00	[15]
	0–100	20.00	5.00	15.00	[1]
	4–80	8.00	NR	NR	[14]
FICTS	0.05–10	0.05	0.42	15.00	This study

LC-FL: Liquid chromatography fluorescence; GC-SIM: Gas chromatography–selected ion monitoring mass spectrometry; LC-MS/MS: Liquid chromatography–tandem mass spectrometry; HPLC: High performance liquid chromatography; UHPLC-FL: Ultra performance liquid chromatography and fluorescence detection; ELISA: Enzyme-Linked Immunosorbent assay; CG-ICTS: Colloidal gold immunochromatographic test strip; FICTS: Fluorescent immunochromatographic test strip. Limit of detection (LOD) is usually defined as the minimum concentration of the component that can be detected with appropriate confidence. 50% inhibition (IC_{50}), the half maximal inhibitory concentration, represents the concentration of an inhibitor that is required for 50% inhibition of things like an enzyme, a cell, a cell receptor or a microorganism. NR: Not reveal.

References

1. Wu, S.W.; Yu, Y.A.; Liu, B.H.; Yu, F.Y. Development of a sensitive enzyme-linked immunosorbent assay and rapid gold nanoparticle immunochromatographic strip for detecting citrinin in monascus fermented food. *Toxins*. **2018**, *10*, e354. doi:10.3390/toxins10090354.
2. Gong, Y.F.; Zhang, M.Z.; Wang, M.Z.; Chen, Z.L.; Xi, X. Development of immuno-based methods for detection of melamine. *Arab. J. Sci. Eng*. **2014**, *39*, 5315–5324. doi:10.1007/s13369-014-1116-5.
3. Zhu, X.X.; Xu, L.; Lou, Y.; Yu, H.N.; Li, X.; Blake, D.A.; Liu, F.Q. Preparation of specific monoclonal antibodies (MAbs) against heavy metals: MAbs that recognize chelated cadmium ions. *J Agric Food Chem*. **2007**, *55*, 7648–7653. doi:10.1021/jf071025l.
4. Marley, E.; Brown, P.; Leeman, D.; Donnelly, C. Analysis of citrinin in cereals, red yeast rice dietary supplement, and animal feed by immunoaffinity column cleanup and lc with fluorescence detection. *J. AOAC Inter*. **2016**, *99*, 1025–1031. doi:10.5740/jaoacint.16-0060.
5. Shu, P.-Y.; Lin, C.-H. Simple and sensitive determination of citrinin in *Monascus* by GC-selected ion monitoring mass spectrometry. *Anal. Sci.* **2002**, *18*, 283–287. doi:10.2116/analsci.18.283.
6. Liu, Y.Q.; Wang, H.W.; Yao, S.; Zhu, P.J. Detection of trace amounts of citrinin in dried orange peel by using an optimized extraction method coupled with ultra-performance liquid chromatography-tandem mass spectrometry. *Biomed. Chromatogr*. **2018**, *32*, e4237. doi:10.1002/bmc.4237.
7. Li, Y.; Zhou, Y.-C.; Yang, M.-H.; Ou-Yang, Z. Natural occurrence of citrinin in widely consumed traditional chinese food red yeast rice, medicinal plants and their related products. *Food Chem.* **2012**, *132*, 1040–1045. doi:10.1016/j.foodchem.2011.11.051.

8. Liu, B.H.; Wu, T.S.; Su, M.C.; Chung, C.P.; Yu, F.Y. Evaluation of citrinin occurrence and cytotoxicity in *Monascus* fermentation products. *J. Agric. Food Chem*. **2005**, *53*, 170–175. doi:10.1021/jf048878n.
9. Liao, C.D.; Chen, Y.C.; Lin, H.Y.; Chiueh, L.C.; Shih, D.Y.C. Incidence of citrinin in red yeast rice and various commercial *Monascus* products in Taiwan from 2009 to 2012. *Food Control* **2014**, *38*, 178–183. doi:10.1016/j.foodcont.2013.10.016.
10. Huertas-Pérez, J.F.; Arroyo-Manzanares, N.; García-Campaña, A.M.; Gámiz-Gracia, L. High-throughput determination of citrinin in rice by ultra-high-performance liquid chromatography and fluorescence detection (UHPLC-FL). *Food Addit. Contam. Part A.* **2015**, *32*, 1352–1357. doi:10.1080/19440049.2015.1055522.
11. Pleadin, J.; Zadravec, M.; Lešić, T.; Vahčić, N.; Frece, J.; Mitak, M.; Markov, K. Co-occurrence of ochratoxin a and citrinin in unprocessed cereals established during a three-year investigation period. *Food Addit. Contam. B.* **2018**, *11*, 20–25. doi:10.1080/19393210.2017.1389994.
12. Vrabcheva, T.; Usleber, E.; Dietrich, R.; Märtlbauer, E. Co-occurrence of ochratoxin A and citrinin in cereals from Bulgarian villages with a history of Balkan endemic nephropathy. *J. Agric. Food Chem*. **2000**, *48*,2483–2488. doi:10.1021/jf990891y
13. Yirga, S. K.; Ling, S.M.; Yang, Y.L.; Yuan, J.; Wang, S.H. The preparation and identification of a monoclonal antibody against citrinin and the development of detection via indirect competitive elisa. *Toxins* **2017**, *56*, 488–495. doi:10.1073/pnas.56.2.488.
14. Kong, D.; Xie, Z.; Liu, L.; Song, S.; Kuang, H. Development of ic-elisa and lateral-flow immunochromatographic assay strip for the detection of citrinin in cereals. *Food Agr. Immunol*. **2017**, *28*, 1–13. doi:10.1080/09540105.2017.1312293.
15. Cheng, H.W.; Yang, Y.; Chen, Y.F.; Chen, X.Q.; Cai, Z.Z.; Du, A.F. Novel monoclonal antibody-based immunochromatographic strip for detecting citrinin in fruit from zhejiang province, china. *PLoS ONE* **2018**, *13*, e0197179. doi:10.1371/journal.pone.0197179.
